# Towards Environmentally Sustainable Diets: Consumer Attitudes and Purchase Intentions for Plant-Based Meat Alternatives in Taiwan

**DOI:** 10.3390/nu14183853

**Published:** 2022-09-17

**Authors:** Han-Shen Chen

**Affiliations:** 1 Department of Health Industry Technology Management, Chung Shan Medical University, Taichung 40201, Taiwan; allen975@csmu.edu.tw; Tel.: +886-4-2473-0022 (ext. 12225); 2 Department of Medical Management, Chung Shan Medical University Hospital, Taichung 40201, Taiwan

**Keywords:** environmentally sustainable food consumption (ESFC), meat alternatives, novel foods, low carbon diet, pro-environmental behavior

## Abstract

With increasing concerns over environmental and animal protection, along with consumers’ preoccupation with health and wellness, the concept of a green diet is gaining popularity. This is leading to a new trend in the food culture of plant-based meat. Employing the extended model of goal-directed behavior (EMGB), this study examines the factors influencing the intentions of young consumers to consume plant-based meat. In particular, this study incorporates two vital constructs in food consumption, namely environmental concern and sensory appeal, into the model of goal-directed behavior (MGB) framework. Data were collected from closed questionnaires: a total of 537 questionnaire responses were gathered in Taiwan. The analysis was performed using the SPSS 25.0 for Windows and AMOS 24.0 for Windows. The results reveal that the EMGB included a satisfactory level of ability in predicting participants’ intentions to consume plant-based meat and was superior to the original MGB. Furthermore, the two incorporated constructs were significant variables influencing consumers’ decision formation. In addition, the attitude, subjective norm, perceived behavioral control, and positive anticipated emotion influenced consumer desire, which, in turn, influenced behavioral intentions.

## 1. Introduction

The Food and Agriculture Organization (FAO) predicts that global meat consumption will increase from 284 million tons in 2007 to 600 million tons in 2050. However, methane emissions from livestock farming are the second-highest source of greenhouse gas emissions after carbon dioxide, and they are a lethal contributor to the greenhouse effect and climate change—a situation that aggravates the ethical and environmental problems associated with meat production [1]. Among the sustainable development goals for the 2030 Agenda launched by the United Nations in 2015, the 12th goal is to “Ensure sustainable consumption and production patterns.” Environmentally sustainable food consumption (ESFC) is defined as “food that meets basic needs and leads to a better quality of life, while minimizing natural resources, toxic material use, and waste throughout its life cycle. and pollutant emissions so as not to jeopardize the needs of future generations” [2]. Major examples of ESFC include focusing on a low-carbon diet [3,4,5]; encouraging and supporting the consumption of local food ingredients [6,7,8]; and promoting organic food [9,10,11]. Through ESFC, consumers can help improve the environment by changing their dietary habits and consumption behaviors.

Given vigorous developments in food technology, new scientific methods are replacing traditional agricultural production and processed food manufacturing globally, for example, the production of plant-based meat [12]. Plant-based meat refers to food products made from plant-based ingredients that replicate the taste, flavor, or appearance of animal meat. For instance, Beyond Meat™ uses soy protein, wheat protein and rice flour to create delicious hamburger meat imitation [13], and Impossible Foods™ uses hemoglobin from the soybean root to replicate the color, texture, and taste of beef, giving the product a fiber structure and appearance similar to that of a real beef burger. Furthermore, Green Monday™ has partnered with FamilyMart, the second-largest convenience store chain in Taiwan, to launch a ready-to-eat food product called “Omnipork Instant Meal Cup” and has collaborated with Taiwan’s largest quick service restaurant (QSR) Bafang Dumplings, selling one million Omnipork dumplings every week. In Asia, in addition to Taiwan, Hong Kong, Singapore, and Thailand, Omnipork has entered China through Alibaba’s e-commerce platform Tmall. According to Euromonitor, the global market for plant-based meat substitutes is expected to expand to approximately USD 16 billion by 2022 [14].

Past studies have focused on determining consumer preferences for different types of products as meat alternatives (e.g., plant-based meat and cultured meat) [15,16]. A major challenge of plant-based meat alternatives is to recreate the appearance, texture, flavor and mouthfeel of meat products [17]. Slade [18] showed that when choosing between beef, plant-based and cultured meat burgers that taste the same, consumers are most likely to choose a beef burger, 21% will buy a plant-based burger and 11% will choose a burger made from “artificial meat”. Michel et al. [19] indicated that consumers are more willing to choose plant-based meat products if plant-based meat alternatives are very similar in taste and texture to real meat.

Bryant and Sanctorum [20] showed that Belgian consumers were significantly more satisfied with their expectations of plant-based meat analogues in 2019 (44%) and 2020 (51%). Boukid et al. [21] indicated that plant-based alternative foods are becoming increasingly popular in the UK. Davitt et al. [22] found that up to 55% of 18–30-year-old students at Iowa State University (ISU) in the Midwest US consume meat analogs. Nearly a third of respondents said they eat as little meat as possible and plant-based alternatives are better for the environment.

As a novel alternative to animal meat, plant-based meat can contribute to ESFC. This is because its production process consumes 46% less energy, produces 90% less greenhouse gas emissions and uses 93% less land and more than 99.9% less water than the production process for beef [23]. If consumers can reduce their consumption of meat by gradually shifting to plant-based meat instead, the global warming phenomenon may be effectively ameliorated. However, plant-based meat products are not well promoted in Taiwan, and the current prices are high. Therefore, it is crucial to understand the key factors attracting consumers, which is the motivation for this study.

The theory of planned behavior (TPB) has been widely used to explore personal behavior in green consumer behavior [24,25,26]. However, Bagozzi [27] noted that while variables such as attitudes, subjective norms, and perceived behavioral control account for the occurrence of a behavior, there is still a lack of explicit motivational variables to explain how intentions are formed to influence specific behaviors. To address the theoretical deficiencies of the TPB, Perugini and Bagozzi [28] proposed the model of goal-directed behavior (MGB) based on the TPB, adding the variables such as desires and anticipated emotions to more thoroughly explain individual psychology, behavioral motivation and actual behavior.

The MGB extends the TPB by adding variables to the model to explain behavioral intentions. The MGB considers the effects of attitudes, subjective norms, and perceived behavioral control on behavioral intentions as described by the TPB, but introduces desire as a mediator of these relationships. In addition, it integrates the relationship between expected emotion and past purchase intention behavior frequency, such as effective process and habit process [28]. Meng and Choi [29] indicated that attitudes, subjective norms, perceived behavioral control and anticipated emotions are antecedents that influence desire and behavioral intention. Lee et al. [30] noted that attitudes and subjective norms have significant positive effects on desire for heritage tourism destinations.

Extant empirical studies revealed that the MGB provides better predictive and explanatory power than the TPB for human decision making [31,32], and it has been widely used for research on leisure and traveling [30,32,33,34,35,36,37], cruises [38,39], mega-sporting events [31,40] and food consumption [41,42,43]. These studies convincingly demonstrated that the MGB has been successfully employed to understand the consumer decision-making process. This study aimed to further contribute to the literature by linking the environmental concern and sensory appeal via the MGB to better understand the decision-making processes underlying the consumer purchases of plant-based meat.

Given the complex relation between environmental concern and the sustainable consumer behavior [44,45], it is imperative to develop additional models to explore the impact of individuals’ environmental concerns on behavioral intentions and their actual behaviors of sustainable consumption [46]. Although scholars have described environmentally sustainable behaviors among consumers [47,48,49,50,51,52], there has been no extensive discussion of the factors triggering pro-environmental consumer behavior in the food industry. Therefore, environmental concern is included as a research variable in this study.

Another variable is the sensory appeal (i.e., appearance, texture and smell of food), which may affect consumers’ intentions toward food consumption in addition to taste or flavor. Baker et al. [53] noted that the sensory appeal influences consumer preference and purchase intention in their choice of food products [54], and Prescott et al. [55] indicated sensory appeal as a key determinant in food purchase and consumption, alongside convenience, quality, safety, price and health. Furthermore, unfamiliarity with novel foods can alter consumers’ sensory expectations and overall liking, which may result in negative expectations [56]. To reduce consumers’ uncertainty about meat analogs, these products are often given taglines, such as “tastes like meat”, so that consumers can relate to their previous experiences and establish good expectations about the characteristics of the product. Therefore, sensory appeal was selected as a research variable in this study.

Past studies regarding meat alternative products have been related to the technical development of novel ingredients [13] or consumer preferences [15,16,19,21]. However, what antecedent variables influence consumers’ desire to purchase plant-based meat? Does desire play a mediating role in consumers’ behavior of purchasing plant-based meat? Owing to inadequate explanations for these questions in the extant literature, there should be further investigation.

According to WorldAtlas [57], Taiwan has the third highest percentage of vegetarians in the world—estimated at approximately 13% the population. The cuisine, which uses traditional and first-generation plant-based meat substitutes such as soy protein, is frequently featured on vegetarian restaurant menus. However, a new generation of plant-based meat substitutes such as Omnipork dumplings are still new to the Taiwanese market.

Nelson et al. [58] noted that young people are the main target customers of the quick service restaurant (QSR). In this study, the participants included young adults (18–30 years old) who had purchased Omnipork dumplings from Bafang Dumpling—Taiwan’s largest QSR. Young adults were selected because they are more concerned about the current environmental situation and more capable of reflecting on their choice of environmentally friendly products relative to their level of judgment [59]. Although the results of convenience sampling may limit the generalizability of the findings, evidence has found that the use of younger populations is reliable for this type of study [60].

This study aimed to identify the factors that influence individuals’ behavioral intention to purchase plant-based meat, using a framework of behavioral theory and empirical research. A more complete integrated model was proposed using the MGB as the basic theoretical framework and adding environmental concern and sensory appeal to the main variables of attitude, subjective norm, perceived behavioral control and anticipated emotion. Subsequently, a questionnaire survey was administered to young adults in Taiwan concerning their attitudes and behaviors toward plant-based meat consumption. The findings are expected to contribute to the analysis and explanation of factors influencing individuals’ behavioral intentions and purchasing plant-based meat, and elucidate how the behavioral theory helps understand the consumption behavior of plant-based meat. Moreover, if managers in the food industry can effectively leverage the crucial determinant factors for consumers’ desire and intention to buy plant-based meat, they can formulate effective strategies by altering the influence of the antecedent variables in the model concerning desire and behavioral intention. Thus, they may increase the effectiveness of marketing practices and application value, which is also a significant contribution of this study.

## 2. Materials and Methods

### 2.1. Research Framework

**Figure 1:**
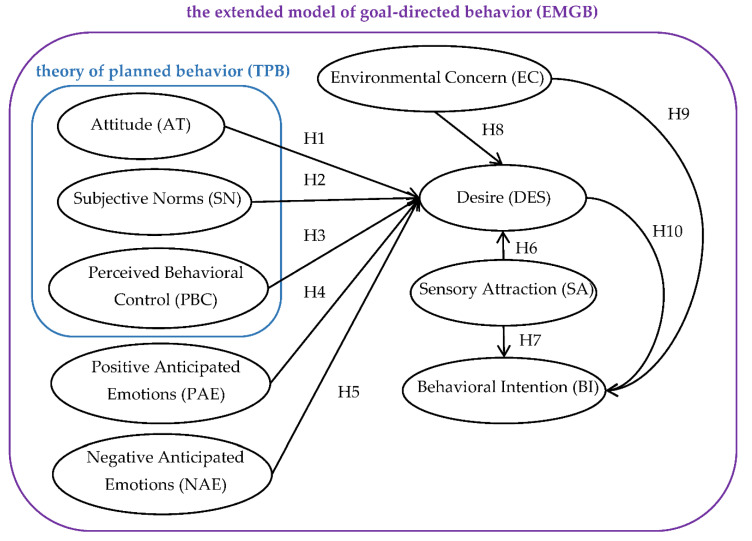
Conceptual framework and study hypotheses.

As discussed in the previous section, this study adopted Perugini and Bagozzi’s [28] MGB as the basic theoretical framework and adds the variables of sensory appeal and environmental concern in the research model to elucidate individuals’ behavioral decisions in purchasing plant-based meat. Based on the literature review, the latent influencing factors of individual consumers’ behavior to purchase plant-based meat are assumed and will be further investigated following the procedures of empirical research. The corresponding research hypotheses are graphically proposed and illustrated in Figure 1.

### 2.2. Research Hypotheses

#### 2.2.1. Relation between Attitude and Desire

Ajzen and Fishbein [61] described that attitudes are mainly composed of the beliefs that an individual forms about an object and their evaluations of the importance of the outcome produced by that behavior to them. Studies have highlighted that attitude is an influencing factor of desire [62,63]. Accordingly, this study proposes the first hypothesis (H1).

**H1.**  *Consumer attitude has a positive impact on desire.*

#### 2.2.2. Relation between Subjective Norms and Desire

Subjective norms refer to the social pressures that individuals may perceive when engaging in a particular behavior by submitting to or considering the opinions of significant others (e.g., family members, close friends and colleagues) [28,64]. Empirical studies have demonstrated that subjective norms influence desire [65,66]. Therefore, H2 is proposed as follows.

**H2.**  *Consumers’ subjective norms have a positive influence on desire.*

#### 2.2.3. Relation between Perceived Behavioral Control and Desire

Perceived behavioral control refers to an individual’s evaluation of their ability or confidence to engage in a behavior; that is, the more the perceived opportunities or resources there are, the fewer obstacles are expected, the higher the perception of control over the behavior is, and the greater the desire may be [28]. Previous studies demonstrated that perceived behavioral control influences desire [65,66,67]. Accordingly, this study proposes H3.

**H3.**  *Consumers’* *perceived behavioral control has a positive effect on desire.*

#### 2.2.4. Relation between Anticipated Emotion and Desire

Previous studies demonstrated that emotions influence consumers’ choice of food and eating behaviors [68,69,70,71]. Lee et al. [72] found that positive and negative anticipated emotions influence positively desire in the context of pop culture tourism. The anticipated emotions that consumers may experience when consuming a product can help us understand their behavioral intentions and behavior toward the product. Williams and Aaker [73] found that individuals’ attitudes are affected when they are exposed to complex emotions. They further demonstrated that the detonation of emotions with duality (e.g., sadness and happiness) is less prone to form an attitude toward their behavior.

Perugini and Bagozzi [28] pointed out that before engaging in a behavior, individuals may form corresponding positive or negative anticipated emotions based on the expected possible outcomes, which in turn, may stimulate or inhibit their desire to engage in the behavior. Several studies have shown that the relation between positive anticipated emotion and desire is positive [65,66,67], whereas others have reported a positive relation between negative anticipated emotion and desire [28,67,74]. Accordingly, this study proposes H4 and H5.

**H4.**  *Consumers’ positive anticipated emotions have a positive effect on desire.*

**H5.**  *Consumers’ negative anticipated emotions have a positive effect on desire*.

#### 2.2.5. Relation between Sensory Appeal, Desire and Behavioral Intention

Sensory attributes are related to the appearance, smell and taste of food and are among the most important factors that consumers consider when making decisions about their eating preference [75,76]. Moreover, the sensory attributes of organic foods, such as taste, color and texture, are associated with pleasure, hedonism, enjoyment and well-being [77,78]. The sensory attributes of food have significantly improved in recent years owing to the advancement in food processing and packaging technologies, motivating consumers to choose food products accordingly [79]. In conclusion, sensory appeal and behavioral intention are correlated. Therefore, H6 and H7 are suggested as follows.

**H6.**  *Consumers’ sensory attraction has a positive effect on desire.*

**H7.**  *Consumers’ sensory attraction has a positive effect on behavioral intention.*

#### 2.2.6. Relation between Environmental Concern, Desire and Behavioral Intention

Environmental concern is defined as individuals’ awareness about environmental issues and the extent to which they exhibit a willingness to contribute to solving them [80]. Consumers who are more concerned about the environment tend to have more positive attitudes toward environmental behavior [81,82]. Pagiaslis and Krontalis [83] indicated that environmental concern have a direct and positive impact on consumers’ behavioral intentions to purchase environmentally friendly products. Furthermore, Yadav and Pathak [84] demonstrated that environmental concern has a significant impact on the behavioral intention of young consumers in developing countries to purchase green products. According to Smith and Paladino [85], consumers who prefer organic products are more likely to engage in environmental activities, reflecting their concern about the environment. Other studies have revealed that environmental concern has a significant positive effect on consumers’ purchase intention to purchase environmentally friendly products [86,87]. Consequently, H8 and H9 are suggested as below.

**H8.**  *Consumers’ environmental concern has a positive effect on desire*.

**H9.**  *Consumers’ environmental concern has a positive effect on behavioral intention.*

#### 2.2.7. Relation between Desire and Behavioral Intention

Behavioral intention refers to the tendency and degree of action with which individuals want to perform a behavior [88]. Studies have validated that desire is the most important antecedent variable of behavioral intention. It has been used to elucidate the formation of behavioral intention [62,65,66] as there is a positive correlation between desire and behavioral intention [29,66,72,89]. Hwang and Kim [90] indicates that desire is an important predictor of intentions to use drone food delivery services.

Furthermore, Lee et al. [74] and Song et al. [67] found that desire plays a key mediating role between the dimensions of MGB (e.g., attitudes, subjective norm, perceived behavioral control, positive anticipated emotions or negative anticipated emotions) and behavioral intentions. Accordingly, H10 is proposed as follows.

**H10.**  *Consumers’ desires have a positive influence on behavioral intentions.*

### 2.3. Questionnaire Design

The questionnaire of this research is designed according to the aforementioned research purposes and relevant literature collection. The back-translation method is used to verify the unintentional distortion of the language to ensure that the meaning expressed by the questionnaire items is consistent with the original scale. Finally, the adjusted Chinese translation version was added to the expert opinion and developed into a pre-test questionnaire. The pre-test questionnaire selected 89 participants for pre-test, and revised the words and sentences of some items to improve the quality of the scale, and then went through reliability and validity. After analysis and grammar adjustment, it was revised into a test questionnaire.

The questionnaire was divided into nine sections. Section 1 was the attitude scale, comprising three items based on Wang et al. [91]. Section 2, regarding the subjective norm, comprised three items based on Han et al. [92]. Section 3 included five items related to perceived behavioral control based on Spash et al. [93] and Han et al. [92]. Then, Section 4 on anticipated emotion included eight items adapted from Bagozzi and Dholakia [94]. Section 5 comprised three items about sensory appeal, adapted from Lee and Yun [54]; Section 6 included five items about environmental concern, adapted from Kim and Choi [95]; and Section 7 had three items concerning desire based on Lee et al. [74]. Section 8 interrogated about behavioral intention with five items based on Chen [96] and Han et al. [92]. Finally, Section 9 was about the participant’s basic information, including gender, religion and diet culture.

In addition to the demographic variables, all questions were administered according to the respondents’ perceptions or actual situation on a 7-point Likert scale from 1 “strongly disagree” to 7 “strongly agree”.

### 2.4. Sample and Data Collection

According to the purpose of this study and hypothesis test, the data were statistically analyzed using structural equation modeling. Wu [97] suggested that the optimal sample size of the structural equation should be determined by the number of questions, and the optimal sample size-to-questions ratio should be between 10:1 and 15:1. Since the questionnaire comprised 35 items, the ideal sample size was 350–525. The data were collected in February and March 2022. A total of 682 questionnaires were distributed, and 613 responses were collected. After excluding 76 invalid questionnaire responses, a total of 537 valid questionnaire responses remained for the analysis, with a recovery rate of 78.7%.

The participants comprised 279 women (52.0%) and 258 men (48.0%). In terms of religious belief, 341 participants followed Buddhism and Taoism (63.5%). With regard to diet culture statistics, 387 participants were nonvegetarians (72.1%) and 150 participants were vegetarians (27.9%).

### 2.5. Data Analysis

This study employed the quantitative research method, and the data were obtained from the questionnaire survey. The data analysis was performed using the IBM SPSS Statistics 25.0 and AMOS version 24.0 statistical packages. This study adopted a two-stage analysis method, the first stage being a confirmatory factor analysis (CFA), and the second stage is based on the overall model fitness analysis. The statistical analysis methods include the descriptive statistics (frequency distribution tables, percentages, means and standard deviations), reliability analysis, validity analysis and structural equation modeling to analyze the causal relations and overall model fit of the hypothesized models and examine the research hypotheses.

## 3. Results

### 3.1. Evaluation of Measurement Models

Since the questionnaire of this research was designed according to the relevant literature collection, CFA was performed to test whether the use of this measurement tool was appropriate for the participants of this study. The details are reported as follows.

#### 3.1.1. Item Reliability

For individual items, the factor loading of potential variables was observed and evaluated. According to Nunnally and Bernstein [98], the standardized factor loading of individual items should be higher than 0.70 and achieve significance. The negative factor loadings of the potential variables for each of the observed items in this study were all above 0.70 and reached the significance level of *p*-value at 0.000, indicating their validity (Table 1).

#### 3.1.2. Internal Consistency

Two indicators were used to examine the internal consistency of the variables: composite reliability (CR) and Cronbach’s alpha values should be greater than 0.7 (0.853–0.947), indicating a high degree of internal consistency of the measured questions for each variable. Bagozzi and Yi [99] suggested that the CR values should be higher than 0.60 to indicate high internal consistency. The CR values in this study are between 0.72 and 0.94, which are significantly higher than the criteria.

#### 3.1.3. Discriminate Validity

As suggested by Fornell and Larcker [100], the criterion of discriminant validity is satisfied if the average variance extracted (AVE) value is greater than the correlation coefficient between the paired variables. As Table 2 reports, the AVE values of all the variables are greater than the correlation coefficients between the variables, denoting the differential validity of the potential variables in this study. Consequently, the questionnaire has a good reliability level.

### 3.2. Overall Model Fitness Verification

The analysis included the overall model fitness and derived the goodness-of-fit index (GFI), adjusted goodness-of-fit index (AGFI), root mean square error of approximation (RMSEA), comparative fit index (CFI), incremental fit index (IFI) and Tucker–Lewis index (TLI). The chi-square fit statistics/degree of freedom (χ^2^/df) in this study model is 2.927, and all results are above the ideal fit standard of 0.90 (GFI = 0.983, AGFI = 0.917, CFI = 0.985, IFI = 0.992 and TLI = 0.942) with an RMSEA of 0.0068, which also meets the judgment criterion of 0.08 or below. Therefore, the overall suitability of the study model is close to good and the study model is valid.

### 3.3. Overall Model Path Analysis

This study employed the structural model analysis to examine the explanatory power of the overall research model. There were 10 path relationships among the nine study variables estimated by the structural equation model, and the values of each path were the standardized coefficients. The results of the analysis indicate that among the 10 research hypotheses in the study model, nine reached the statistically significant levels (*p* < 0.05). Only H5 could not be supported.

The results of the hypothesis validation are presented in Table 3. The variables in the integrated model, including attitude (β = 0.245, *p* < 0.001), subjective norms (β = 0.278, *p* < 0.001), perceived behavioral control (β = 0.072, *p* < 0.001), positive anticipated emotions (β = 0.254, *p* < 0.001), sensory appeal (β = 0.192, *p* < 0.001) and environmental concern (β = 0.405, *p* < 0.001) significantly and positively influence individuals’ desire to purchase plant-based meat. Therefore, H1, H2, H3, H4, H6 and H8 are supported. However, the effect of negative anticipated emotion (β = −0.018, *p* > 0.05) does not reach the significance level; therefore, H5 is not supported. Among the various factors, “environmental concern” has the highest impact on “desire”, followed by “subjective norm” and “positive anticipated emotion”—in that order.

Among the predictive factors of “behavioral intention”, desire (β = 0.396, *p* < 0.001), environmental concern (β = 0.375, *p* < 0.001) and sensory appeal (β = 0.172, *p* < 0.001) significantly and positively influence individuals’ intention to purchase plant-based meat. Therefore, hypotheses H7, H9 and H10 are supported. In terms of impact, “desire” has the highest predictive power on “behavioral intention”, followed by “environmental concern” and “sensory appeal” in that order.

In general, all hypotheses are supported barring H5; that is, individuals’ desire to purchase plant-based meat is influenced by variables such as attitude, subjective norms, perceived behavioral control, positive anticipated emotions, sensory appeal and environmental concern. When an individual has a more positive attitude, stronger support from significant others, better behavioral control and higher positive anticipated emotions, sensory appeal and environmental concerns, the person is more likely to have a stronger desire. Furthermore, a stronger desire to buy encourages the individual’s intention to buy.

## 4. Discussion

The findings demonstrate a significant and positive relation between attitude and desire, corresponding to the findings of previous studies [62,101,102]. In the MGB, attitudes are indirectly influenced by behavioral intentions through desires. That is, individuals evaluate the potential benefits or losses of engaging in a particular behavior to determine whether to push the desire. When an individual’s evaluation of the anticipated outcome of a behavior is positive, the individual has a stronger desire to perform the behavior [28]. Second, subjective norms present a significant positive effect on desire, which is consistent with the findings of previous studies [66,101,102] and Perugini and Bagozzi’s [28] discussion of the causal relationship between goal-oriented behavior patterns, both confirming the positive relation between subjective norms and desire. This implies that when individuals perceive that their significant others or groups (e.g., family members or peers) are supportive of their purchase of plant-based meat, their desire to purchase plant-based meat increases. In conclusion, perceived behavioral control exerts a significant positive effect on desire. This finding is consistent with previous studies [28,66,101], suggesting that individuals’ desire to purchase plant-based meat may be enhanced if they have sufficient time, money or opportunity to do so.

Furthermore, the results of this study indicate that positive anticipated emotions have a significant positive effect on desire, implying that the more positive (i.e., happy and pleasant) an individual’s anticipated emotions are about buying plant-based meat, the stronger their desire to buy plant meat is. This is consistent with past studies that reported a positive relation between emotions and desires [28,66,101,102]. Moreover, the finding that sensory appeal has a significant positive effect on desire is consistent with the findings of past research [54,103,104]; that is, when plant-based meats replicate the taste and texture of meat and are attractively priced, they are most likely to successfully replace meat. The more positive the sensory appeal is, the more positive the consumer’s attitude is toward the plant-based meat product. The results of the current analysis also confirm that environmental concern is a significant influence on desire and behavioral intention, as found by previous researchers [86,87]. In other words, consumers’ environmental concern affects their behavioral intentions toward plant-based meat products.

Furthermore, this study demonstrates that desire should positively and significantly influence individuals’ behavioral intentions, suggesting that the greater the desire is, the stronger the individual’s willingness to purchase plant-based meat is likely to be. Moreover, the validation model in which desire is the most important antecedent variable of behavioral intentions is highly suitable for explaining the generation discourse of behavioral intention [28]. Meanwhile, desire plays a mediating role between attitude, subjective norm, perceived behavioral control, positive anticipated emotion, sensory appeal and behavioral intention. This finding is similar to the results of other research [62,66,101] and is aligned with the view that only desire can mediate intention in the MGB. Perugini and Bagozzi [88] highlighted that desire is the most important antecedent variable of behavioral intention. Therefore, all aforementioned findings support the conclusion of a positive influence relation between desire and behavioral intention in this study.

Since desire is not impacted by negative anticipated emotions, the H5 is invalid. Researchers have noted that individuals should engage in specific behaviors to avoid negative anticipated emotions [28,67,102]. However, judging from the context of this study, if individuals are unable to consume plant-based meat, they may also experience the anticipated negative emotions of sadness, depression or anxiety.

This study has some limitations. First, the participants are primarily young people aged 20–30 who have previously purchased plant-based meat in Taiwan; therefore, the extrapolation of the findings is limited. To improve the accuracy of the research results and obtain complete information, subsequent researchers can use this study as a basis and collect data from various other market segments (e.g., different age groups, regions and cities) to increase the coverage of the sample. The extrapolation, accuracy and reference value of the research results can be enhanced, which will be helpful for food industry managers to formulate marketing strategies for target markets and improve the research on the factors influencing the behavioral intention of novel foods.

Subsequent researchers should consider including lifestyle and personality traits in the MGB as segmentation variables and subsequently examine whether the behavioral models and relationship structures of the segmented groups with different lifestyle or personality traits are different so that food marketers can make strategic plans for different segmentations. Furthermore, because meat consumption is strongly influenced by culture and religion [105], follow-up studies should analyze the perceptions and opinions of consumers from different cultural backgrounds (e.g., Muslims) regarding plant-based meat products to increase the applicative value of research in practice.

In addition, future studies could perform random sampling collection and use larger sample sizes, as well as consider other variables such as word-of-mouth recommendations, type of food, health awareness, or food safety, to explore whether these factors influence consumers, thereby consummating the research framework.

## 5. Conclusions

On the basis of the model validation results, the study discusses the following findings and proposes the following conclusions and recommendations.

First, the integrated model has a good fit. Second, perceived attitudes, subjective norms, perceived behavioral control, positive anticipated emotions, sensory appeal and environmental concern positively influence individuals’ desire to purchase plant-based meat. Finally, individuals’ behavioral intention to purchase plant-based meat is positively influenced by factors such as desire, environmental concern and sensory appeal.

The findings establish that consumers’ attitudes, subjective norms, perceived behavioral control and environmental concern have significant impacts on behavioral intentions toward plant-based meat. Consequently, it is advised that companies in the food or dining industries should emphasize the corporate environmental social responsibility by conducting seminars and media publicities to elucidate that artificial meat products are beneficial to the environment because of their lower carbon footprint compared with meat such as pork and chicken. Recently, the number of vegetarians has increased owing to consumers’ desire for environmental protection, carbon emission reduction and personal health and wellness. Hence, it is suggested that food or dining industry operators promote vegetarian tourism and courses to expand the business opportunities of their products and contribute to accomplishing the ultimate goal of carbon reduction and ESFC.

## Figures and Tables

**Table 1:** Reliability summary of the measurement model.

Variable/Item	Mean	Standard Deviation	Standardized Factor Loading	Average Variation Extracted (AVE)	Composite Reliability (CR)
Attitude (AT)	5.84	0.83		0.65	0.87
1. It is a good idea to consume plant-based meat products.	5.78	0.72	0.865 ***		
2. It is a wise choice to consume plant-based meat products.	5.90	0.69	0.828 ***		
3. I like the idea of consuming plant-based meat products.	5.84	0.92	0.943 ***		
Subjective norms (SN)	4.99	1.11		0.75	0.92
1. Most people who are important to me think that I should buy plant-based meat products.	5.20	1.07	0.903 ***		
2. Most people I value would prefer to buy plant-based meat products.	4.86	0.92	0.892 ***		
3. The degree of influence from an individual or group strongly influences my decision to purchase plant-based meat products.	4.91	1.31	0.885 ***		
Perceived behavioral control (PBC)	5.69	0.72		0.83	0.94
1. I am willing to pay more for plant-based meat to protect the environment.	5.76	0.69	0.923 ***		
2. I believe that plant-based meat products can improve the surrounding environment.	5.89	0.71	0.917 ***		
3. I would definitely choose plant-based meat in a fast-food restaurant.	4.45	1.32	0.906 ***		
4. I think it is ideal to buy plant-based meat products.	5.72	1.09	0.931 ***		
5. I can decide for myself whether to choose plant-based meat products.	6.62	0.54	0.952 ***		
Positive anticipated emotions (PAE)	5.69	0.62		0.58	0.72
1. I would be excited if I could go to a fast-food restaurant next month and eat plant-based meat products.	5.78	0.58	0.706 ***		
2. I would be happy if I could go to a fast-food restaurant next month and eat plant-based meat products.	5.62	0.61	0.712 ***		
3. I would be delighted if I could go to a fast-food restaurant next month and eat plant-based meat products.	5.65	0.72	0.725 ***		
4. I would be satisfied if I could go to a fast-food restaurant next month and eat plant-based meat products.	5.72	0.65	0.718 ***		
Negative anticipated emotions (NAE)	3.21	1.12		0.72	0.81
1. I would be sad if I couldn’t go to a fast-food restaurant next month to eat a plant-based meat product.	3.21	1.07	0.702 ***		
2. I would be depressed if I couldn’t go to a fast-food restaurant next month to eat a plant-based meat product.	3.46	1.28	0.725 ***		
3. I would be upset if I couldn’t go to a fast-food restaurant next month to eat a plant-based meat product.	3.13	1.15	0.705 ***		
4. I would be anxious if I couldn’t go to a fast-food restaurant next month to eat a plant-based meat product.	3.02	1.03	0.711 ***		
Sensory appeal (SA)	5.48	0.72		0.82	0.78
1. Plant-based meat looks good.	5.69	0.65	0.921 ***		
2. Plant-based meat has a good texture.	5.43	0.81	0.906 ***		
3. Plant-based meat looks delicious.	5.36	0.73	0.917 ***		
Environmental concern (EC)	6.58	0.57		0.86	0.92
1. I am very concerned about the status of the world environment and what it will mean for my future.	6.02	0.42	0.924 ***		
2. Humans are severely damaging the environment.	6.75	0.37	0.931 ***		
3. When humans interfere with nature, the consequences are often devastating.	6.81	0.35	0.975 ***		
4. The natural balance is delicate and can easily be disrupted.	6.53	0.48	0.919 ***		
5. Human beings must live in harmony with nature.	6.78	0.55	0.924 ***		
Desire (DES)		1.08		0.75	0.89
1. I’m looking forward to visiting a fast-food restaurant next month to eat a plant-based meat product.	5.04	1.22	0.802 ***		
2. I want to visit a fast-food restaurant next month to eat a plant-based meat product.	5.11	1.19	0.792 ***		
3. I wish to visit a fast-food restaurant next month to eat a plant-based meat product.	5.18	1.29	0.736 ***		
Behavioral intention (BI)	5.28	0.88		0.82	0.93
1. If plant-based meat is available, I will try to buy plant-based meat products.	5.43	0.87	0.865 ***		
2. If I can choose again, I would still buy plant-based meat products.	5.17	0.94	0.842 ***		
3. I consider myself a loyal customer of plant-based meat products.	5.12	1.17	0.846 ***		
4. I will recommend friends and family to buy plant-based meat products	5.29	0.85	0.825 ***		
5. Even though the price of plant-based meat is higher, I would still buy it.	5.38	0.94	0.831 ***		

Note: *** < 0.001.

**Table 2:** Correlation coefficient and the AVE square root of measurement model.

Dimension	1	2	3	4	5	6	7	8	9
1. AT	***0.806***								
2. SN	0.528	***0.866***							
3. PBC	−0.041	0.079	***0.911***						
4. PAE	0.683	0.468	−0.076	***0.762***					
5. NAE	0.315	0.294	0.023	0.304	***0.849***				
6. SA	0.296	0.184	−0.062	0.392	0.205	***0.906***			
7. EC	0.626	0.494	0.035	0.515	0.327	0.316	***0.927***		
8. DES	0.817	0.674	0.069	0.758	0.396	0.524	0.796	***0.866***	
9. BI	0.605	0.583	0.027	0.525	0.362	0.469	0.748	0.859	***0.906***

Note: The values in the diagonal cells (in bold and italic) are the square root of the AVE of the potential variables.

**Table 3:** Results of the overall path analysis of the model.

Hypothesis	Path Relationship	β Path Coefficient	t-Value	Hypothesis Test
H1	AT→DES	0.245 ***	6.328	Supported
H2	SN→DES	0.278 ***	7.932	Supported
H3	PBC→DES	0.072 ***	2.757	Supported
H4	PAE→DES	0.254 ***	7.293	Supported
H5	NAE→DES	−0.018	−0.482	Not supported
H6	SA→DES	0.192 ***	6.716	Supported
H7	SA→BI	0.172 ***	4.547	Supported
H8	EC→DES	0.405 ***	10.493	Supported
H9	EC→BI	0.375 ***	6.688	Supported
H10	DES→BI	0.396 ***	7.592	Supported

Note: *** < 0.001.

## Data Availability

The data that support the findings of this study are available from the corresponding author, H.-S.C., upon reasonable request.
